# Did Alexander Fleming Deserve the Nobel Prize?

**DOI:** 10.1007/s11948-019-00149-5

**Published:** 2019-10-31

**Authors:** Martin Sand

**Affiliations:** Department of Values, Technology and Innovation, Faculty of Technology, Policy and Management, TU Delft, Jaffalaan 5, 2628 BX Delft, The Netherlands

**Keywords:** Serendipity, Penicillin, Moral luck, Praiseworthiness, Desert, Control, Reward

## Abstract

Penicillin is a serendipitous discovery *par excellence*. But, what does this say about Alexander Fleming’s praiseworthiness? Clearly, Fleming would not have received the Nobel Prize, had not a mould accidently entered his laboratory. This seems paradoxical, since it was beyond his control. The present article will first discuss Fleming’s discovery of Penicillin as an example of moral luck in science and technology and critically assess some common responses to this problem. Second, the Control Principle that says that people are not responsible for things beyond their control will be defended. An implication of this principle is that Alexander Fleming’s desert, which is based on his epistemic skills, remains untouched by luck. Third, by distinguishing different notions of praiseworthiness, a way to resolve the paradox of moral luck will be elaborated. Desert provides only a *pro tanto* reason to determine whether someone is an appropriate addressee of reward. Here, luck can make a difference. Forth, it will be argued that stimulating the quest for socially beneficial science provides a compelling reason to treat scientists with equal desert differently. Penicillin provides striking evidence for the importance of this quest and showcasing it incentivizes the making of socially beneficial science. Ultimately, it will be justified why Fleming deserved the Nobel Prize in at least one sense of the concept.

## The Problem of Luck and its Significance

On place seven of their list of the “Top 10 Nobel Prize Controversies”, the TIME magazine mentions Alexander Fleming’s reception of the award in 1945 (Philipps 2011). In a short note, TIME’s author Jak Philipps points out that a similar type of antibiotic than the one described by Fleming in his famous article “On the antibacterial action of cultures of a penicillium, with special reference to their use in the isolation of B. Influenzae” from 1929, has allegedly already been discovered in the late 19^th^ century, thus questioning the novelty of Fleming’s findings.^1^ Philipps also underlines that Fleming admitted that “the discovery was a complete accident.” Indeed, throughout his life Fleming frequently alludes to this judgement as several of his colleagues confirmed (Macfarlane 1984, p. 271). However, the TIME’s article concludes that Fleming is “worthy of high praise” because the discovery of Penicillin “has since saved millions of lives worldwide […].” Similarly surrounded by an air of paradox, Joan Bennet and Kim-Thong Chung write that “luck played a big part in gaining [Fleming’s] place in the scientific pantheon.” On the same page, they state that Fleming “himself is an enduring role model […] for success in clinical research.” (Bennett and Chung 2001, p. 182) Does this make sense? How can someone deserve high praise and receive the most prestigious award in science for a discovery that has been considered an accident by himself and others? Fleming seems to be a beneficiary of a universal inconsistency: an inconsistency in judging people’s desert, where luck is supposed to play no role, and our factual ways of crediting and appraising, where luck makes a huge difference, exemplarily prevailing in the previously presented judgments of Fleming’s praiseworthiness.

In the present article, I will explore the case of Alexander Fleming as one of a seemingly morally lucky scientist *par excellence*: Fleming received praise and rewards for something that was partially beyond his control. One might insist that despite the obvious vicinity of this case to the moral luck problem, it is not Fleming’s moral but his *scientific* worth that is at stake here. This is true and alludes to an important distinction for various discussions in philosophy of technology. However, I will not elaborate on the differences between moral and scientific virtues, because they are of minor relevance for the present issue [regarding this distinction, see (Davis 2012, pp. 796 f., 801)]. The unsettling problem of moral luck suggests that neither of those types of virtues are the sole basis of credit and reward. Instead, factors beyond people’s control also affect those reactions and this challenges widespread intuitions about people’s responsibility.

While this paper has a strong focus on the case of Fleming’s discovery of Penicillin and his reception of the Nobel Prize, the following insights also advance the philosophical debate on moral luck. In line with my approach, many philosophers emphasize the ambiguity of blameworthiness. Many of them also stress that considerations regarding someone’s desert is one but not the only basis for determining the type and quality of reward or sanction (Concepcion 2002, p. 456; Enoch and Marmor 2007, p. 412 f.; Zimmerman 2002, p. 567). However, none of these authors articulate in much detail what else ought to be considered aside from moral desert when determining the appropriate quality of sanction or reward. The Incentive-Argument that will be outlined in the present paper is exemplary for such type of consideration and, thus, fills an important gap in the moral luck debate. At the same time, Fleming’s discovery of Penicillin is the ideal backdrop to demonstrate the more general implications of this problem for contemporary sanction and reward systems in science and technology.

Hence, the issue of Fleming’s Nobel Prize is not just a philosophical conundrum. As many ingredients of Fleming’s case can be found in other areas of science and technology, luck seems to threaten the legitimacy of science and technology policies that utilize prizes as incentives and blame and sanction as deterrence more generally: Can it ever be justified that people receive credit for scientific or technological achievements as luck regularly enters their making (Friedman 2001, p. 271; van de Poel and Sand 2018)? It is, therefore, no wonder that scholars from other fields such as Responsible Research and Innovation (RRI) have expressed worries regarding luck’s central role in science and technology (Grinbaum and Groves 2013, p. 139; Stilgoe et al. 2013, p. 1569). Taking these worries seriously, I will first present the case of Fleming, outline how luck was involved in the discovery of Penicillin and discuss his scientific merits. In the following section, I will explore the problem of moral luck in more detail and shed some common objections up-front. I will distinguish different types of luck that have been involved in the discovery of Penicillin and defend the Control Principle that says that agents are morally appraisable only for things within their control. That Alexander Fleming’s moral standing remains untouched by luck is an implication of this principle. By distinguishing different notions of blameworthiness, I will show that there can be good reasons for differential treatment of lucky and unlucky scientists with equal moral standing. Before summarizing my results, I will argue that such justified differential treatment is not unfair. A good reason for differential treatment of equally virtuous scientists is to incentivize the valuable quest for socially beneficial science. Ultimately, this paper defends the rationale behind the Nobel Prize.

Before presenting Fleming’s case in more detail, I wish to shed two famous objections against the Nobel Prize in its current form up-front. Elisabeth Crawford criticises that the Nobel Prize does not cover any of the more recent, applied disciplines, which might have an even bigger effect on society (to the good and bad): civil engineering, infrastructure, energy, information technologies and so on (Crawford 1998). Furthermore, Crawford suggests in line with other notable authors that Nobel Prizes, which are currently rewarded to three or less individuals, fall short of the collective nature of modern scientific endeavours (Casadevall and Fang 2013; Friedman 2001, p. 273; Merton 1968, p. 56).^2^ Both points express legitimate criticism of the current shape of the Nobel Prize. However, they do not undermine the *general idea* behind such award. Luck, on the contrary, might do so. Even if the Nobel Prizes were rewarded to bigger groups or whole institutions from both basic and more applied sciences, luck interferes with collective efforts as much as with individuals. Luck is also omnipresent in applied and basic research. These facts would remain deeply unsettling, even if the Nobel Prize were substantially modernized in these regards.

## The Case of Alexander Fleming

Alexander Fleming was born in 1881 in Ayrshire, Scotland and raised together with seven siblings by his father. With a scholarship and a small legacy from an uncle he attended St. Mary’s Medical School in London where Almroth E. Wright made notable advancements in vaccine treatment of infectious diseases at the time. After his graduation in 1906, Wright became his scientific mentor and Fleming was soon renowned as “one of Sir Almroth Wright’s most enthusiastic followers.” (Bennett and Chung 2001, p. 165) The first time Fleming is reported to having been scientifically lucky was in 1921, when he discovered that his nasal fluid showed lytic activity towards a bacterium, which he initially termed “A.F. coccus” (later *Micrococcus lysodeikticus*). While Fleming claimed that the bacteria were harboured in his nasal fluid from a traditional cold, his former laboratory assistant Victor Allison suggests that it was a chance contaminant from air or dust (Macfarlane 1984, p. 100). In the following years, together they proved the presence of such bacteriolytic agents in tears, skin, tissue and many other animal and plant compounds. Despite the publication of these findings in the *Proceedings of the Royal Society* in 1922 and in later publications, this research received initially very little recognition similar to Fleming’s more famous work on Penicillin. The remarkable series of events that happened in 1928 and after, is a continuous source for scientific imagination and awe. Even scholars who are prepared to face the unpredictable workings of luck, must be baffled by these events. I shall quote a passage from Gwyn McFarlane’s Fleming-biography, which provides a masterly account of luck’s quintessential role in the discovery of Penicillin:

[…] the whole chain of chance events involved in the discovery [of Penicillin] has an almost unbelievable improbability. […] First, Fleming inoculates a plate with staphylococci and it happens to become contaminated with a rare, penicillin-producing strain of mould. Second, he happens not to incubate this plate. Third, he leaves it on his bench undisturbed while he is away on holiday. Fourth, the weather during this period is at first cold and then warm. Fifth, Fleming examines the plate, sees nothing interesting and discards it, but by chance, it escapes immersion in lysol. Sixth, Pryce [one of Fleming’s former colleagues] happens to visit Fleming’s room, and Fleming decides to show him some of the many plates that had piled up on the bench. Seventh, Fleming happens to pick the discarded penicillin plate out of the tray of lysol (in which it should have been immersed), and on a second inspection sees something interesting. (Macfarlane 1984, pp. 247–248)

Penicillin affects bacterial growth by inhibiting their cell division. It was, therefore, of greatest significance that the petri dishes inoculated with staphylococci were exposed to cold temperatures at first. A dish fully covered with bacteria could not have been contaminated by the penicillin-mould in the first place. Also, the characteristic inhibition of growth-pattern could not have occurred, had the temperature in the room not changed soon after. Bennett and Chung conclude that “although it is relatively easy to demonstrate bacterial inhibition, the bacterial lysis that caught Fleming’s attention on the famous Petri Plate could only have been due to an extremely unusual set of events.” (Bennett and Chung 2001, p. 168) Although, Fleming published the results in the *British Journal of Experimental Pathology* in 1929, they remained largely unnoticed until some 10 years later, when luck struck again. Fleming himself, despite having used Penicillin successfully at least once in 1932 (Wainwright 1987, p. 43), seemed to have lacked clear evidence for its therapeutic potential (Macfarlane 1984, p. 253). It remained up to the research team of Howard Florey and Ernest Chain from the William Dunn School of Pathology in Oxford to pick up his work in 1938. Successful experiments with animals were undertaken a year later (Chain et al. 1940), early successful clinical applications with humans followed soon after. As war was heading towards the British peninsula, Florey and Chain decided to undertake further experiments and to scale up Penicillin production in the United States. Through cooperation of scientists, private companies and government agencies, they soon managed to scale up *Penicillium chrysogenum*, a higher yielding stream of Penicillin. In 1945, Fleming, Florey and Chain obtained the Nobel Prize for Physiology and Medicine, while Fleming received by far the most media recognition in the following years. Although he accepted the publicity with little resistance, he hesitated to consider himself worthy of the Prize. William van Heyningen, a former colleague, reports: “[Fleming] told me often that he didn’t deserve the Nobel Prize, and I had to bite my teeth not to agree with him.” (Macfarlane 1984, p. 271) Nevertheless, regarding his scientific qualities McFarlane concludes unambiguously that Fleming “had two great aptitudes–the power to see what was really there and the more mysterious flair for distinguishing between the important and the trivial–whether, in fact, what he was seeing was the tip of a vast, submerged iceberg or merely a passing ice-floe.” (p. 262) Despite doubts about his dedication (p. 270), one must acknowledge Fleming’s tenaciousness in undertaking tedious laboratory work for several decades. Throughout these years, he constantly trained his vision to detect relevant anomalies, which made him “the perfect eyewitness for this unlikely microbiological contingency […] a receptive observer.” (Bennett and Chung 2001, p. 168) What it means to be a skilled observer varies with the context: In this case, it presumes knowledge of the state of the art of the scientific discipline and an idea of what counts as relevant and novel from this perspective (Copeland 2017). McFarlane summarizes Fleming’s scientific merits in a similar manner: “If [Fleming] cannot be given the credit for the accidents, he should be granted the keen eye that observed them and the prepared mind that appreciated their interest.” (Macfarlane 1984, p. 265)

## Moral Luck and Common Objections

The previously presented narrative of Penicillin’s luck-soaked discovery is a commonplace. From this and other instances of serendipitous discoveries, Nicholas Rescher concludes that “luck is not only a prominently operative factor in such explicitly chancy matters as gambling or risk-taking entrepreneurship but […] it also plays a significant role in such thoroughly rational enterprises as scientific inquiry.” (Rescher 1995, p. 91 f.) This verdict is shared by many. Mike Martin states that “[s]erendipity is ubiquitous in science.” (Martin 2007, p. 52)^3^ A large and thrilling list of anecdotes of serendipitous discoveries and accidental technological advancements is presented by Royston M. Roberts, of course, featuring Penicillin (Roberts 1989). The involvement of luck is a central and unpredictable factor in science and technological development. What then is the problem of *moral* luck?

Luck’s ubiquity in science raises a normative issue concerning the praiseworthiness of lucky scientists like Fleming. The most striking concern is desert in its relation to the fairness of rewards such as the Nobel Prize. In Alfred Nobel’s will, it is determined that the Prizes ought to be given “to those, who shall have conferred the greatest benefit to mankind.” (Nobel 1895) Thus, it is obvious that without luck’s contribution, Fleming would not have received the Nobel Prize, since he would not have conferred to the benefit of mankind to such an extent. This raises the question whether he actually deserved it. With regard to scientific prizes, the issue translates into one about fairness: Is it justifiable that he instead of other scientists with similar skills and determination got the Prize, as luck constitutes the only difference between them? Although, I focus here on an example in which someone’s degree of praiseworthiness is at stake, cases concerning *blameworthiness* are more frequently discussed in the moral luck debate. For the sake of simplicity, I assume that the following arguments apply to both praise- and blameworthiness and their corresponding responses reward and sanction.^4^ A famous case that exemplifies moral luck and suggests its existence in ordinary morality is the drunken driver case:

If someone has had too much to drink and his car swerves on to the sidewalk, he can count himself morally lucky if there are no pedestrians in its path. If there were, he would be to blame for their deaths, and would probably be prosecuted for manslaughter. But if he hurts no one, although his recklessness is exactly the same, he is guilty of a far less serious legal offence and will certainly reproach himself and be reproached by others much less severely. (Nagel 1991, p. 29)

In this passage, Thomas Nagel describes how practices of punishment (prosecution for manslaughter) and moral appraisal are amended in the light of consequences which the drunken drivers did not ultimately control. This is the case despite a deeply ingrained common sensical belief that people are not blameworthy for things beyond their control. Nagel concludes that this is paradoxical (Nagel 1991, p. 34). First, it must be pointed out that Nagel’s exposé is sketchy and unassertive regarding what exactly is affected by luck: a person’s guilt, his blameworthiness, his legal liability, or all of them? Second, even if we agree that in ordinary morality moral luck exists and makes a difference for how people usually judge and treat one another in the way suggested by Nagel, this does not imply that this is right. My response is twofold: First I’ll argue that luck *should* not make a difference for *moral appraisal*. Second, I will show that it *can* make difference when determining the quality of sanction or reward. This is not paradoxical and not always unfair. Before I outline my position in more detail, I shall respond to two common objections to moral luck that suggest that the phenomenon is epistemically reducible or insignificant as long as there is some degree of control. I begin by discussing the latter.

Picking up the core of a celebrated aphorism by Louis Pasteur, Michael Pritchard argues that some “[e]ngineers […] seem to be somehow *prepared to be lucky*. That is, because of their skills and commitment, they are prepared to pick up cues and run with them, to notice what others fail to notice, and so on.” (Pritchard 2001, p. 398)^5^ In this vein, one might argue that Fleming’s scientific skills, his power of observation and eager experimentalism, initially made him aware of the mould in the petri dish and it is those skills that induce praise and admiration. One might argue that he received the Nobel Prize for having successfully trained those skills and not for the accidental occurrence of a substance later known as Penicillin. Affirmatively, I said that the discovery of Penicillin was *partially* beyond his control and the previously listed skillset is that part which was within his control. With this proposal, Pritchard responds to the question whether discoveries in engineering are *just* matters of luck (p. 398). Pritchard answers to the point: “No, not *just*.”

Obviously, driving drunk increases the likelihood of swerving on the sidewalk and producing fatal accidents as much as attentive and considerate laboratory work increases the likelihood of making discoveries. Through their actions, agents make *a partial contribution* to the course of events, thereby, often implicitly acknowledging their impact on the likelihood of certain events’ occurrence. This acknowledgment of one’s contribution is usually a ground to evaluate attentive- or recklessness behaviour as praise- and blameworthy respectively (Sand 2018, pp. 68–69).^6^ In this manner, it is suggested that developing certain epistemic skills increases the likelihood of becoming scientifically lucky, such as training certain virtues increases the likelihood of becoming morally lucky (Copeland 2017; McKinnon 2014). In so far as it is up to the agent to deliberately train such skills or accept certain risks when driving negligently, these freely adopted volitions constitute the basis of someone’s moral standing (Concepcion 2002, p. 459). However appealing, Pritchard’s advance in Pasteurian spirit does not resolve the moral luck problem. Recall that Nagel suggests in his example that we make a difference between two *equally* reckless drivers. With regard to scientific honours, Fleming would not have received the Nobel Prize despite being equally observant and experimentally skilled, had he not additionally been lucky. The paradox emerges as the differential assessment and treatment of two (hypothetically) *equally prepared* scientists, purely based on things beyond their control. Emphasizing the importance of certain controllable features of an agent–such as scientific skills or preparedness–misses the point, since luck seems to be the crucial tipping point regarding her degree of laudability and worthiness of scientific reward.

Also, assuming that the moral luck problem is merely epistemic does not bring us further towards a solution. Rescher makes such claim when writing: “The morally lucky culprit is lucky not because his moral condition is superior but simply he is not unmasked. The difference here is not moral but merely epistemic.” (Rescher 1995, p. 154)^7^ In this manner, the lucky driver can call himself lucky, because without a victim he will not be caught. Later on, Rescher underlines this point: “The ‘morally lucky’ villain is not, in fact, *morally* lucky (by hypothesis, he is a villain), but is *socially* lucky only because that reprehensible nature is not disclosed to the community.” (p. 155) Obviously, luck is often epistemic. Luck affects whether or not a misdeed is detected. Consider two persons A and B, who continuously drive without a ticket. One day, A gets caught by the conductor and receives a fine. The epistemic argument suggests that B would receive the same fine, would she be caught, which suggests that her luck is merely epistemic. However, not all forms of luck are merely epistemic in this sense (Concepcion 2002, p. 457). Nagel’s example suggests that some luck is moral. *Ex hypothesis*, we know what both drunken drivers did (driving drunk) and what factually happened: Either was lucky and the other one was not and hit a child. Ignorance cannot stop the paradox from emerging here, because it is absent by design. The epistemic argument suggests that equally vicious people would receive the same degree of blame and sanction, if their misdeed came to light. But people who are caught driving drunk without hitting anybody are neither prosecuted for manslaughter nor are they blamed to the same degree as people, who hit somebody. This shows that moral luck cannot always be reduced to epistemic luck.

In this section, I introduced the moral luck problem through the drunken driver case and argued that this problem also appears in the context of scientific rewards. It was shown that both the epistemic argument and Pritchard’s “Pasteurian-argument" cannot settle the worries raised by moral luck. Thus, I will propose a different way of approaching the paradox in the following sections.

## Different Types of Luck and a Defence of the Control Principle

First, it is important to distinguish several types of luck, because Fleming seems to have benefitted from different types of luck, each requiring a different response. Let us begin with *constitutive luck*, as Nagel calls it (Nagel 1991, p. 33). Dana Nelkin outlines constitutive luck as follows: “Since our genes, care-givers, peers, and other environmental influences all contribute to making us who we are (and since we have no control over these) it seems that who we are is at least largely a matter of luck. Since how we act is partly a function of who we are, the existence of constitutive luck entails that what actions we perform depends on luck, too.” (Nelkin 2013b) In this sense, Fleming was born with a basic set of attitudes, maybe with a natural curiosity and attraction to the experimental sciences. If those attitudes were beyond his control, why praise him for them? Firstly, it should be emphasized that many philosophers concede that there is a certain influence on one’s character (Hanna 2014, p. 694; Sand 2018, p. 167; Smith 2005). When Nagel introduces constitutive luck, he writes ambiguously “[t]o some extent such a quality may be the product of earlier choices; to some extent it may be amenable to change by current actions.” (Nagel 1991, p. 33) That there is a certain extent to which a character is amenable, is a crucial concession, even if it remains largely a matter of luck. That it is not *completely* a matter of luck, is in fact the only concession needed. Secondly, it should be pointed out that scientific skills such as those for which Fleming received most praise by biographers and contemporaries–his acute, unbiased power of observation and a keen eye to expect the unexpected in an experiment (Macfarlane 1984, p. 262)–are hardly the qualities that one can inherit. An inherited scientific curiosity does not help one telling apart a dirty petri dish from one that contains an important antibacterial substance. These skills have to be learned and continuously trained, which requires diligence and effort (Copeland 2017; McKinnon 2014).

More significant than constitutive luck is *resultant moral luck* characterized by Nagel as luck “in the way things turn out” (Nagel 1991, p. 28), which is most strikingly present in the drunken driver case. Another often discussed example of resultant luck introduces the two assassins George and Georg: both attempt to murder Henrik, but Georg’s bullet is caught by a (massive) bird passing its way, while George succeeds murdering him (Zimmerman 2002, p. 560). In Fleming’s case, resultant luck enters the story when the Oxford group surrounding Florey and Chain pick up his work on Penicillin and prove its efficacy in clinical trials. Their research obviously affected the clinical impact of Fleming’s initial findings and defied his control entirely. Without other scientists’ contribution, his scientific achievements would have been far less momentous.^8^ Obviously, Fleming’s research has also been affected by the *circumstances* that occurred during his summer holidays in 1928.^9^ If the mould had remained absent from the petri dish, he would not have had the opportunity to discover Penicillin in the first place, and hence, this discovery could not have resulted in medical revolution. Nagel calls this type of luck *circumstantial moral luck* and it obviously has a close connection to *resultant moral luck* (Hanna 2014, p. 695; Nagel 1991, p. 34). Circumstantial luck is often discussed with reference to the following case: Judge A would freely accept a bribe, if someone *would* offer him one. So would judge B, but only B gets one offered and actually accepts it (Hartman 2016, p. 2847).

Returning now to the paradox of moral luck, one might argue that Fleming’s *moral standing* is unaffected by the workings of luck. In this manner, I claim that he would be equally praiseworthy, had he not discovered Penicillin, because whatever his praiseworthy qualities they are exactly the same from this counterfactual viewpoint. This also suggests that he is just as praiseworthy as other actual scientists with the same epistemic skills and scientific virtues. Similarly, George and Georg have the same “mark on their moral ledger” (Concepcion 2002, p. 456), despite one of them succeeding and the other one failing because of luck. These claims are implications of the Control Principle (CP) to which a majority of philosophers in the moral luck debate appeal (Concepcion 2002, pp. 459–460; Enoch and Marmor 2007, p. 407; Zimmerman 2002, p. 560). I have touched upon this principle above as a deeply ingrained conviction of common-sense morality, because Nagel introduces it in this way (pp. 25–27). It might be more fundamental than the term “common sense” suggests. It certainly has enormous intuitive appeal (Enoch and Marmor 2007, p. 407). The standard formulation of CP is as follows (Nelkin 2013b):

CP We are morally assessable only to the extent that what we are assessed for depends on factors under our control.

Following this formulation, luck, which is presumably opposed to control (Enoch and Marmor 2007, p. 407), is indeed “irrelevant for moral responsibility.” (Zimmerman 2002, p. 559) When claiming that Fleming is just as morally laudable as his hypothetical counterpart, who does not discover Penicillin, I presume that CP entails the idea that it would be mistaken to assess two people who are equal in all regards differently, if the results of their actions (or their actions) differ due to luck’s workings. This means, that a judge who *would* freely accept a bribe but never gets one offered is as blameworthy as one who freely accepts a bribe and also that both assassins George and Georg are equally blameworthy although only one of them succeeds with murdering Henrik, because they develop the same evil plan and attempt to execute it equally determined. Hence, I adhere to the following corollary of CP, which is CP_C_:

CP_C_ Two people ought not to be morally assessed differently if the differences between them are due only to factors beyond their control.

CP and CP_C_ mutually entail each other. The concept of control features large in both principles, despite being contentious. If control is understood narrowly as the irrevocability of a process instigated by an agent, then very few things are within our control, maybe only our volitions (Khoury 2018). Even bodily movements are not necessarily within our control in this sense, as an epileptic seizure can interfere with acting at any time. However, living together in societies were hardly possible, if our actions would systemically fail for such reasons. For the present context, it is sufficient to note that people control their volitions and their actions at least partially, as suggested above, by directly influencing the likelihood of succeeding or failing.

While many philosophers find the principles CP and CP_C_ intuitively appealing, they have not escaped criticism. The most recent and sophisticated criticisms of CP and its corollary have been brought forward by Nathan Hanna and Robert Hartman (Hanna 2014; Hartman 2016). Discussing their objections will strengthen my position and help to understand and alleviate some difficulties accompanied with the kind of counterfactual reasoning employed before. Hanna defends an example of circumstantial luck which supposedly directly undermines CP:

Jimmy promised his spouse to stop eating at the local McDonald’s. But he knows that the following is true.

M If Jimmy were driving by the local McDonald’s while it is open, he would succumb to temptation and break his promise.

Jimmy is intent on not breaking his promise and he exploits his knowledge of M towards this end. He regularly avoids driving by the McDonald’s while it’s open. Call these circumstances c. Does M make Jimmy culpable to some degree? If so, to what degree? To the first question, I say no because Jimmy deliberately avoids c to avoid breaking his promise. […] To me at least, it seems inappropriate to take him to be culpable on the basis of M, partly because of his intentions and behaviour with respect to c. […] Even if my claim that M doesn’t make Jimmy culpable to same degree were false, though, this wouldn’t entail anything specific about how culpable M makes him. I’d still deny that M makes him as culpable as a promise-breaker. (Hanna 2014, p. 686)

Later, Hanna reaffirms his conviction that it seems particularly implausible “to say that [Jimmy] is as culpable as a promise-breaker […].” (Hanna 2014, p. 688) For the following reasons, I do not find this counterexample convincing.^10^ Hanna asks, whether M makes Jimmy culpable to some degree, assuming that those who oppose circumstantial moral luck must affirm this. Hanna is convinced that if Jimmy would drive by the McDonald’s and, thus, break his promise despite his deliberate effort to avoid this from happening, defenders of CP must assert that he is just as culpable as a promise-breaker. If one denies—as I do—that circumstances make a difference for moral assessment, one has to affirm this. But this is far from obvious. Compare this to the judges case: Even though B never acts upon his intent to accept a bribe, because he never gets one offered, he can be considered just as wicked as A, who accept the offered bribe. Judging them equally bears on the fact that they entertain the same malicious intent to accept a bribe. Circumstances M, in contrast, are not something for which Jimmy is culpable. Hanna’s description suggests that Jimmy’s behaviour under circumstances M is rather compulsive: Driving by the McDonald’s necessitates that he will be eating there. Unlike the judges, who deliberately entertain malintent, Jimmy’s behaviour—like the behaviour of addicts—is beyond his control and no one is culpable for things beyond control. Therefore, advocates of CP do not have to regard Jimmy as culpable as a promise-breaker. Thus, the example is in relevant respects different from the judges case, where circumstances provide an opportunity to actualize a malicious intent and, hence, do not matter for moral assessment. Jimmy does not act upon bad intentions under circumstances M: He is determined by them. This suggests more generally the ambiguity of the concept “circumstance”: Some circumstances are better conceived as *opportunities*, others as *determinants*. In some circumstances, for instance, when one’s life is threatened, almost everybody would perform morally dubious actions. But that does not taint everybody morally dubious, because circumstances which leave one without reasonable alternatives excuse the behaviour in question (Fischer and Tognazzini 2011, p. 388 f). Such circumstances deprive moral assessments of their fundament. It is rather dubious of Jimmy that he makes a promise, of which he knows he cannot keep, if certain circumstances occur. Judging him on this ground, however, is independent from circumstances M.

Hanna continues his criticism with another direct attack on CP. He argues that assessing people’s real moral standing on the basis of counterfactual reasoning has counterintuitive implications (Hanna 2014, p. 688). He recalls an example from Thomas Nagel (Hartman 2016, p. 2850; Nagel 1991, pp. 33–34), whose core idea is essentially this: We would all act very differently, if circumstances were different from those in which we are coincidentally, actually living in. People who are currently considered as ordinary and mild-mannered would become collaborators of an evil regime and contribute to genocide, if they had grown up in a political dictatorship such as Nazi Germany. This is certainly possible and defies individuals’ control. If counterfactuals like this are the basis of moral appraisal, then most people’s moral standing is quite different from how it is ordinarily assumed to be. This is counterintuitive: We do not think of mild-mannered people as having the same moral standing as regime collaborators.

While it is plausible that many of us would collaborate with an evil regime, we do not know exactly who would. This epistemic limitation puts a halt to Hanna’s argument before any counterintuitions to CP even emerge. Generally, one can assume that possible worlds must be sufficiently similar to the actual world in order to allow for a plausible moral assessment *across them*. Assuming that we would act very differently in counterfactual worlds, does not entitle us to draw a conclusion about *how* differently we would act. In contrast, the possible world in which Georg’s bullet is hindered by a bird is similar enough to entitle us to draw the same conclusion about his moral blameworthiness than about his actual sibling George who kills Henrik. Also judges A and B are conceived in such way, that one can safely assume that both *would* accept bribes. Here, epistemic limitations do not undermine judgments based on CP and CP_C_. Regarding counterfactual worlds that are fundamentally different than ours, a person’s moral standing remains necessarily opaque (Enoch and Marmor 2007, p. 422 f.). Despite its limited scope, this is where Rescher’s epistemic argument has a proper place. The restriction to sufficient similarity between counterfactual worlds neither undermines CP nor CP_C_. In fact, presuppositions about true counterfactuals are prior to moral assessments and, therefore, pose a menace not only for deniers of moral luck. Counterfactuals are an essential part of our metaphysics of agency, which makes determinism so dubious in the first place (Keil 2007, pp. 118–119). An ascription of responsibility for an action presupposes that the agent could have done something different, if only omitting the act. The agent is evaluated in light of these alternatives, which are presumably true counterfactuals.

In this section, I have defended the Control Principle, which is the view that people’s moral standing is unaffected by luck despite contrary inclinations in ordinary contexts. Fleming would be morally praiseworthy to the exact same degree, had he not discovered Penicillin. This is dubious, because he would still be *treated* differently. He would not have received the Nobel Prize. This will be discussed in more detail in the following section.

## The Incentive-Argument and the Ambiguity of Blame- and Praiseworthiness

The arguments presented, underscore that there is no such thing as *moral luck*. However, it is undeniable that even if Alexander Fleming is judged as being equally praiseworthy according to CP had he not discovered Penicillin, it is obvious that he would not have received the Nobel Prize. As mentioned before, the Nobel Prize is rewarded for scientific *achievements* and not for doughty but unsuccessful attempts to make them. Obviously, the paradox of luck is still looming large: In a sufficiently similar counterfactual world in which Fleming does not come across the speck of mould in his laboratory, he has the same marks on his moral ledger. The same moral appraisal is adequate. Why is it then that in terms of worthiness of a prize, he deserves more in the actual world in which he makes the finding than in the counterfactual world in which he does not?

Can such differential treatment despite stemming from matters beyond control be justified? Differential treatment due to luck is obviously also existent in legal contexts: Even if we agree that both reckless drivers have the same moral standing, one hesitates to accept that both of them (or none–which would be an equal treatment, too) deserve ending up in jail. As mentioned before, drunken drivers are not prosecuted for manslaughter. Hence, there is *legal luck* (Enoch 2010). However, these forms of punishment and reward do not stand in a paradoxical tension to CP and CP_C_, because they concern *different meanings* of blameworthiness. It is indeed defensible and, therefore, not unfair that luck makes a difference regarding some forms of sanction and reward. This will be shown in the remainder of this paper. It is noteworthy that the problem of fairness, although it seems to motivate concerns about moral appraisal in the moral luck debate from the word go, appears first time in the context of *differential treatment* of people with the same moral standing (Hieronymi 2004; Nelkin 2013a, p. 119). However, it is meaningless to speak of an unfair assessment of someone’s moral standing. A person’s moral standing is simply what it is. A judgment about it can be adequate or right, but not fair or unfair (Hanna 2014, p. 693). Thus, the issue of fairness concerns the differential treatment of actual people and *other actual* people who have been less lucky despite the same virtues: Why not awarding another epistemically skilled and diligent scientist with the Nobel Prize? Would such person not deserve it, too? Contrasting people with their lucky or unlucky counterfactuals, as done before, only exacerbates this basic problem of fairness.

Praise- and blameworthiness are ambiguous concepts (Coates and Tognazzini 2012). Recall Nagel’s discussion of the drunken drivers, where he writes that their *responsibility* and their *guilt* differ. He adds that one of them is worthy of *more blame* and *reproach* and that he committed a more serious *legal offence* and *deserves worse sanction* than the other (Nagel 1991, p. 29). The ordinary concept of blameworthiness covers all (and even more, e.g. reactive attitudes, resentment, bad conscience) of these meanings and so it seems paradoxical that someone’s blameworthiness is and is not affected by luck. But as argued before, a person’s moral standing is indeed not affected by luck or in other words, luck should not affect our judgments about moral standings. However, this principle (CP) does not imply that blame- and praiseworthiness in terms of sanction and reward, cannot be affected by luck. In an agreeable manner, David Concepcion writes:

By making proper use of these distinctions, we can see that there is nothing paradoxical about concluding that the unlucky driver both is and is not responsible for the child’s death. He is responsible in different sense of the term ‘responsibility.’ For example, we may claim that the unlucky driver is legally liable in some sense without concluding that he is morally liable or appraisable. He may justifiably be fined or imprisoned by the state, even if he does not owe an apology. Even if we suppose that he is morally liable in some sense, we may still hold that he is not blameworthy. He may owe expressions of comfort and regret even if he is not blameworthy. (Concepcion 2002, pp. 456–457)

How can things beyond control make a difference for other forms of responsibility such as legal liability, prize-worthiness or overt blame? Consider the following example: A person P is sitting with her child in a tram and mistreats the kid in a minor way, for instance, by raising her voice after the kid has smudged its shirt with chocolate. We assume, for the sake of argument, that such behaviour is morally wrong and reflects her character in a relevant sense. Conclusively, the fault is attributable to her and “stains” her moral ledger. Imagine, however, that there are only strangers around and, thus, no one possesses the *moral authority* to translate P’s blameworthiness into an overt judgment or outward blame. Consider, in contrast, another person P*, who mistreats her child in the same manner while her best friend is around. Her best friend will condemn her behaviour and ask her to calm down: All kids are occasionally smudgy, hence, there is no reason to make a fuzz. Can P* claim to having been treated unfairly, as P did exactly the same and the presence of her friend was not within her control? No: While P and P* are equally blameworthy, there is a compelling reason why strangers restrain themselves with overt blame against P. In contrast to P*’s best friend, strangers lack the relevant moral authority in this case.

I derive from this and other examples discussed in the literature on appropriate blame (Coates and Tognazzini 2012; Smith 2007) that responses (e.g. sanction, overt blame, or reward) to blame- or praiseworthy behaviour are deserved or fair, if there is a *good reason* to install such responses.^11^ Fairness understood as a consideration about the equal treatment of people with the same moral standing does not exhaust the variety of good reasons that are significant here. Retribution, restoration, rectification of damage, deterrence and encouragement are goals that can play a central role, too. Determining the quality of these goals and their specific weight belongs to the realm of moral theory and it is beyond the scope of this papers to establish a full-fletched account of their relation (Smith 2007, p. 476). It does not follow from this proposal that any response that would be conducive to societal welfare such as punishing innocent people is morally acceptable, which is an often-articulated criticism of such practically oriented idea of liability to blame (Wallace 1994, p. 94). In other words, this theory of blameworthiness does not force us to adopt a type of consequentialism (Vargas 2007, pp. 158–159). If examples about punishing the innocent are persuasive, they show that consequentialist reasons are not necessarily good reasons and fall, therefore, outside of the scope of this theory. Whether these examples are persuasive is of minor importance here. The distinction between my account of blameworthiness and consequentialism should merely be underscored.

Good reasons can muster a consideration about someone’s moral standing as suggested in the previous section and additional considerations, for instance, about the restoration of justice (legal liability: drunken driver), the rectification of damage, the significance of harm that has been done and the relation between the doer and the blamer (moral authority) (Coates and Tognazzini 2012, p. 204; Enoch and Marmor 2007, p. 413). In general, considerations about someone’s moral standing are underdetermined regarding the appropriateness of a certain response (Smith 2007, p. 477 f.): Having a negative mark on one’s moral ledger does not necessarily imply a specific (or, in fact, any) sort of sanction or punishment. In this manner, blame- and praiseworthiness provide only *pro tanto* reasons to treat people in certain ways.

How does this proposal translate to the question of fairness in terms of being worthy of a prize? Aside from Fleming’s qualities and skills such as his tenaciousness and power of observation, the obvious societal impact of Penicillin adds another reason to justify a certain response, since it is clear evidence for the beneficial effects of scientific discoveries on mankind. The award showcases these effects and, thus, encourages the pursuit of socially beneficial research (Strevens 2006, p. 165).^12^ As mentioned before, the general idea behind awards in science is steering science into socially desirable directions: They are a form of research policy (van de Poel and Sand 2018). Prizes are supposed to encourage and incentivize desirable research and the best way to achieve this is to link them directly to such desirable research, lucky or not. This is not limited to Nobel Prizes. Harriet Zuckerman’s research on stratification in science suggests that “[rewards in science] serve much the same purpose as they do in other institutional spheres. They validate past performance and **provide a degree of motivation for the future** [own emphasis]. They bring attention to performance judged to be of high quality, thereby reinforcing the standards by which performance is to be assessed.” (Zuckerman 1970, p. 252)

One might object to this and argue that a fairer procedure repudiating luck utilizes a lottery to determine Nobel Prize Laureates. One could imagine a well-stirred bowl containing scraps of paper with the names of the most virtuous and skilful scientists of the year (Needless to say that establishing such list is quixotic, which indicates another opening for Rescher’s epistemic argument (Partha and David 1994, p. 499)). Then someone successively takes three scraps out of the bowl and the names on them denote the Nobel Prize winners. This procedure disregards any accidental discoveries succeeding someone’s research and considers only what scientists can control: their virtues and skills. It also gives all of them the same chance of winning and does, thereby, justice to their equal desert (Broome 1990, p. 97 f.).

Note, that this procedure adds even more chance to the equation. It suggests that if you are a tenacious and skilful scientist you might make a great discovery with the support of luck and with some *additional* luck you might also receive the Nobel Prize. In contrast, the direct linkage advocated before suggests that you might make a great discovery with the help of luck and get the Nobel Prize *for it.* A random procedure cannot motivate one to strive for socially beneficial discoveries, because it does not increase the likelihood of receiving a prize as you need an additional portion of luck.^13^ Hence, it makes no difference, whether you strive for it or not. In this sense, what seems to be the fairest procedure in terms of luck’s absence is less reasonable in light of the important goal of steering science in a socially desirable direction. If I am right, then it is not the fairest procedure.

## Conclusions and Outlook

In the present article, I discussed Penicillin as a serendipitous discovery *par excellence*, yielding Alexander Fleming prizes and fame. Furthermore, I introduced the problem of moral luck as the concern about the appropriate degree of his praiseworthiness, since the discovery was partially beyond his control. Conflicting intuitions regarding this spawn the paradox of moral luck. After distinguishing different forms of luck involved in Fleming’s work, I have defended the Control Principle, the view that people are blameworthy only for things within their control. However, I also explained that blame- and praiseworthiness are ambiguous concepts sometimes used merely to denote moral laudability and sometimes used to refer to a variety of responses to moral appraisal such as sanction, praise and reward. In the light of these distinctions, it is not paradoxical to assume that somebody is blameworthy in one of these senses but not in another. Against this backdrop, I asked again whether Fleming deserved the Nobel Prize. His virtues and epistemic skills put a positive mark on his moral (or, if you prefer, scientific) ledger. However, in this sense his desert is no bigger than that of other equally skilful, but lesser fortunate scientists. This is a direct implication of CP_C_. As equal desert gives rise to claims for equal treatment, the more pressing concerns are centred around the notion of fairness. I argued that desert understood as moral standing provides only *pro tanto* reasons for determining adequate treatment. Aside from desert, broader societal needs, for instance, provide further reasons to be acknowledged here. In this deviated sense, Fleming deserves the Prize more than other, unlucky scientists. His findings are a clear evidence for the increased likelihood of making momentous discoveries, if one pursues science with the necessary attentive- and tenaciousness.^14^ Showcasing these results as worthy of great reward has an incentivizing function, which it would not have were the Prize distributed *randomly* amongst the most skilled and virtuous scientists. While such procedure appears to be fairer at first sight, since it renunciates resultant luck, it undermines motivating scientists to pursuit great scientific achievements. This can be considered unfair for being negligent of the important goal of furthering the benefit of mankind.

As an outlook, I will shed a critical light on two underexposed issues in the neighbourhood of my arguments. Above, I presupposed that the Nobel Prize has an incentivizing effect on scientists, a presupposition that could be questioned. Are scientists motivated by the prospect of receiving the Nobel Prize? There are intriguing narratives of yearlong races between scientists in their pursuit of making a Nobel worthy discovery before their rivals (Wade 1978a, b). But not only on the individual level is there great evidence for the motivating power of this reward. Since it has been established, the Prize “allowed nations to prove their right to honor by showing their ability to make supreme contributions to the collective advance of civilization” (Friedman 2001, p. 268), a challenge which many nations readily accepted (Hansson 2018a). Today, “China’s obsession” with the Nobel Prize as much as Japan’s attempts to produce more Nobel Prize winners (e.g. by sending researchers abroad and funding more basic research) are clear evidence for its incentivizing power on both the individual and the national level (Cao 2014, p. 141; Low 2001, p. 42).

Finally, one might question just how objective or unbiased the decision-making procedure of the Nobel Prize Committee actually is. What counts as the “greatest benefit to mankind” is a contentious matter that constitutes a constant source of struggle for the Nobel Committee (Friedman 2001, p. 274). The discussion about Nobel’s own legacy is a great example for such equivocality (Bucchi 2018; Hansson 2018b). However, the vagueness of the term “beneficial to mankind” is defensible, because it gives justice to the typical dynamics of societal value changes. “Beneficial to mankind” nowadays contains the values safety, sustainable and transparency, values that were of lesser importance at the beginning of the last century. Nobel’s original formulation is adaptable to such value changes. Furthermore, the Committee’s past tendency to regard more theoretical advancements in contrast to experimental or practical research as most “beneficial to mankind”, is anything but arbitrary as some authors suggested (Friedman 2001, p. 274; Luttenberger 1996; Zuckerman 1996). Consider the unambiguous pessimism of historian Franz Luttenberger, who identifies the Committee’s previous decisions with games of chance:

It would be naive to believe that the Nobel prize honours each year’s most meritorious scientist. The selection process follows its own logical and dynamics. *Justice*, or *the perfect choice* are notions that are sometimes far less applicable to Nobel prize matters than *coincidence* or the *game of chance*. (Luttenberger 1996, p. 238)

In a similar manner, Robert Friedman narrates the history of the Nobel Prize with a human, a fallible face and writes that the list of winners was neither “natural nor inevitable.” Instead choosing winners has entailed “judgment and volition, compromise and insistence.” (Friedman 2001, p. 267) It is important to note that judgment and volition are nothing equal to luck, although it might look like it for those who are or favoured or missed by it.^15^ In fact, they are quite the opposite when it comes to controlling their occurrence. Here, the responsibility, the obligation for critical self-assessment rests on the Nobel Committee. If–as Friedman charges the Committee on another page of his narrative (p. 44)–personal bias and misjudgement can be found throughout the history of the Nobel Prize, this does not undermine its just nature as defended before, just as the existence of bribed referees does not undermine the *rationale* behind soccer games. Even if genuine luck, which cannot be controlled by anyone, is involved in choosing Laureates, my arguments remain intact. The possibilities of luck’s interference are infinite and every game in a non-ideal world such as ours might suffer from it: Recommendation letters can get lost, potential advocates of someone’s research suffer an accident and so on. This, however, does not undermine the Incentive-Argument, as training to become a better soccer player does not become meaningless, just because an even perfectly placed shot can end up hitting the goalpost. Training still increases the likelihood of winning next time.

## Footnotes

1 This assumption is contested as “[v]irtually all contemporary writers concede that Alexander Fleming deserves full credit for the scientific discovery of penicillin.” (Bennett and Chung 2001, p. 171) Milton Wainwright notes that “while it is clear that […] Victorian scientists observed antagonism between fungi and bacteria, we have no way of knowing whether they observed the penicillin effect sensu stricto.” (Wainwright 1987, p. 43) A more critical view on this issue is advanced by Robert Crease (1989).

2 As a fourth actor in the discovery of Penicillin, Norman Heatley was a “key figure” of the Oxford group (Bennett and Chung 2001, p. 172). He did not receive the Prize and, hence, his contribution is largely forgotten. The same applies to “Mouldy Mary”, Miss Mary Hunt, who discovered the mold that produced *Penicillium crysogenum*, an important step towards the large-scale production of Penicillin (Copeland 2018; Macfarlane 1984, p. 211).

3 A terminological note might be due here: Luck is a wider concept than serendipity, which is confined to accidental discoveries. Luck can be understood as a property of the world, as an event without preceding causes. Such event cannot be foreseen. But, even if there is no genuine luck in this sense, even if determinism is true, there would be events that cannot be foreseen, because of our epistemic limitations (Coffman 2007; Rescher 1995). P is lucky regarding an event E, if E is unexpected, significant and beyond P’s control, while the latter is most significant for moral luck (Enoch and Marmor 2007, p. 407). Note, that one cannot consider P lucky for choosing a job against previous assurances to never switch jobs. Although P’s decision is unexpected as it stands against previous inclinations, it is within her control. Obviously, events that are uncontrollable for some people are not necessarily uncontrollable: P’s being chosen as a winner is uncontrollable by P, but not by the committee that choses the winner. If P is unexpectedly chosen and the award is significant, she can count herself lucky.

4 It has been argued that unlike punishing people undeservingly, rewarding people undeservingly is not unfair, at least not to them (Watson 2004, p. 284). This might be true, but it does not imply that it is fair to reward two equally deserving people differently. The unequal treatment constitutes a problem of fairness that appears in both directions (Browne 1992, p. 354; Enoch and Marmor 2007, p. 424).

5 “In the field of experimentation, chance only favours the prepared mind.” Quoted from Bennett and Chung (2001, p. 167).

6 I am very grateful to an unknown reviewer, who made me aware of an ambiguity in my original depiction of this issue.

7 In his repudiation of moral luck, Rescher’s view is similar to mine. He writes that unlucky people’s “moral *record* may differ, but their moral *standing* does not.” (Rescher 1995, p. 154) On the next page, he goes on: “Most recent discussions of ‘moral luck’ fail to appreciate the fact that the opportunity-deprived immoralist’s good luck appertains not to his moral *status*, but merely to his *reputation*.” I merely doubt that all cases of moral luck can be reduced to epistemic luck.

8 It has been argued that serendipitous discoveries are emergent properties of such scientific networks (Copeland 2017).

9 A list of the different types of contingencies that affected the rise of modern science is presented by Trout (2016, 2019). Trout suggests that these contingencies led scientists in early modern Europe to theories, which turned out to be “sufficiently good” (2016, p. 105). His list includes: 1. Psychological contingencies, 2. environmental contingencies, 3. contingencies of timing, 4. sweeping effects of simple historical contingencies, 5. unintended cultural side effects, 6. biological contingency and cognitive limits. Some of these types could be subsumed under the category circumstantial luck (1, 2, 3, 4) in Nagel’s terminology, others under the category resultant (5), and constitutive luck (6).

10 I am very grateful to an unknown reviewer, who helped me to clarify my response to this challenging example.

11 Brad Hooker calls this broad understanding of fairness “all things considered moral rightness.” He rejects this idea, because there are other terms to express this idea better, such as morally best, most morally desirable or morally right (Hooker 2005, p. 332). The term fairness should be reserved to relate only to a subset of moral reasons, namely desert, agreements and needs (p. 350). This is not a particularly forceful objection against my broad understanding. More importantly, if socially beneficial science is understood as a societal need, my proposal is in complete harmony with Hooker’s narrower account of fairness.

12 It is important to emphasize that it is really the *pursuit of socially beneficial science* that should be encouraged with the relevant epistemic skills being a necessary but insufficient condition for achieving this. Take an analogy: One does not only want to encourage people to *intend* to save children, which is supposedly the only thing they can completely control (Khoury 2018, p. 1376). One rewards people for saving children, because one wants children to be saved.

13 It should be obvious that the other two equalizing options–giving everyone prizes or not giving anyone prizes–also lack motivational power. Why would one try to run faster in a sprint competition, if eventually everyone wins no matter how slow?

14 In his award ceremony speech, Göran Liljestrand underlined that “[i]n a time when annihilation and destruction through the inventions of man have been greater than ever before in history, the introduction of Penicillin is a **brilliant demonstration** [own emphasis] that human genius is just as well able to save life and combat disease.”

15 This also applies to Toby Sommer’s notion “Bahramdipity”: The suppression of new knowledge by an established representative of a reigning scientific paradigm (Sommer 2001).

## References

[CR2] Bennett JW, Chung K-T (2001). Alexander Fleming and the discovery of penicillin. Advances in Applied Microbiology.

[CR3] Broome J (1990). Fairness. Proceedings of the Aristotelian Society.

[CR4] Browne B (1992). A solution to the problem of moral luck. The Philosophical Quarterly.

[CR5] Bucchi M (2018). “The winner takes it all?” Nobel laureates and the public image of science. Public Understanding of Science.

[CR6] Cao C (2014). The universal values of science and China’s nobel prize Pursuit. Minerva.

[CR7] Casadevall A, Fang FC (2013). Is the Nobel Prize good for science?. The FASEB Journal.

[CR8] Chain E, Florey HW, Gardner AD, Heatley NG, Jennings MA, Orr-Ewing J, Sanders AG (1940). Penicillin as a chemotherapeutic agent. The Lancet.

[CR9] Coates DJ, Tognazzini NA (2012). The nature and ethics of blame. Philosophy Compass.

[CR10] Coffman EJ (2007). Thinking about luck. Synthese.

[CR11] Concepcion DW (2002). Moral Luck, control, and the bases of desert. The Journal of Value Inquiry.

[CR12] Copeland S (2017). On serendipity in science: Discovery at the intersection of chance and wisdom. Synthese.

[CR13] Copeland, S. (2018). “Fleming leapt on the unusual like a weasel on a vole”—Challenging the Paradigm of Serendipity in Science. *Perspectives on Science* (Forthcoming).

[CR14] Crawford E (1998). Nobel: Always the winners, never the losers. Science.

[CR15] Crease RP (1989). Righting the antibiotic record. Science.

[CR16] Davis M (2012). A plea for judgment. Science and Engineering Ethics.

[CR17] Enoch D (2010). Moral Luck and the Law. Philosophy Compass.

[CR18] Enoch D, Marmor A (2007). The case against Moral Luck. Law and Philosophy.

[CR19] Fischer JM, Tognazzini NA (2011). The physiognomy of responsibility. Philosophy and Phenomenological Research.

[CR20] Friedman RM (2001). The politics of excellence: Behind the nobel prize in science.

[CR21] Grinbaum A, Groves C, Owen R, Bessant JR, Heintz M (2013). What is “Responsible” about responsible innovation? Understanding the ethical issues. Responsible innovation.

[CR22] Hanna N (2014). Moral Luck defended. Noûs.

[CR23] Hansson N (2018). Anmerkungen zur wissenschaftshistorischen Nobelpreisforschung. Berichte zur Wissenschaftsgeschichte.

[CR24] Hansson N (2018). What’s so special about the Nobel Prize?. Public Understanding of Science.

[CR25] Hartman RJ (2016). Against luck-free moral responsibility. Philosophical Studies.

[CR26] Hieronymi P (2004). The force and fairness of blame. Philosophical Perspectives.

[CR27] Hooker B (2005). Fairness. Ethical Theory and Moral Practice.

[CR28] Keil G (2007). Willensfreiheit.

[CR29] Khoury AC (2018). The objects of moral responsibility. Philosophical Studies.

[CR30] Low M (2001). From Einstein to Shirakawa: the Nobel Prize in Japan. Minerva.

[CR31] Luttenberger F (1996). Excellence and chance: The Nobel Prize Case of E. von Behring and É. Roux. History and Philosophy of the Life Sciences.

[CR32] Macfarlane G (1984). Alexander fleming: The man and the myth.

[CR33] Martin MW (2007). Creativity: Ethics and excellence in science.

[CR34] McKinnon R (2014). You make your own luck. Metaphilosophy.

[CR35] Merton RK (1968). The Matthew effect in science. Science.

[CR36] Nagel T (1991). Moral Luck. Mortal questions.

[CR37] Nelkin DK (2013). Desert, fairness, and resentment. Philosophical Explorations.

[CR38] Nelkin, D. K. (2013b). Moral Luck. In E. N. Zalta (Ed.), *The Stanford Encyclopedia of Philosophy* (Winter 2013 ed.). https://plato.stanford.edu/archives/win2013/entries/moral-luck/: Metaphysics Research Lab, Stanford University.

[CR39] Nobel, A. (1895). Will. Retrieved 20.08.2018 https://www.nobelprize.org/alfred-nobel/full-text-of-alfred-nobels-will/

[CR40] Partha D, David PA (1994). Toward a new economics of science. Research Policy.

[CR41] Philipps, J. (2011). Top 10 Nobel Prize Controversies. Retrieved 20.08.2018, from TIME Magazine http://content.time.com/time/specials/packages/article/0,28804,2096389_2096388_2096384,00.html

[CR42] Pritchard MS (2001). Responsible engineering: The importance of character and imagination. Science and Engineering Ethics.

[CR43] Rescher N (1995). Luck: The brilliant randomness of everyday life.

[CR44] Roberts RM (1989). Serendipity: Accidental discoveries in science.

[CR45] Sand M (2018). Futures, visions, and responsibility: An ethics of innovation.

[CR46] Smith AM (2005). Responsibility for attitudes: Activity and passivity in mental life. Ethics.

[CR47] Smith AM (2007). On being responsible and holding responsible. The Journal of Ethics.

[CR48] Sommer TJ (2001). Suppression of scientific research: Bahramdipity and Nulltiple scientific discoveries. Science and Engineering Ethics.

[CR49] Stilgoe J, Owen R, Macnaghten P (2013). Developing a framework for responsible innovation. Research Policy.

[CR50] Strevens M (2006). The role of the Matthew effect in science. Studies in History and Philosophy of Science Part A.

[CR51] Trout JD (2016). Wondrous truths: The improbable rise of modern science.

[CR52] Trout JD, Church IM, Hartman RJ (2019). Luck in science. The Routledge handbook of the philosophy and psychology of luck.

[CR53] van de Poel I, Sand M (2018). Varieties of responsibility: Two problems of responsible innovation. Synthese.

[CR54] Vargas M, Fischer JM, Kane R, Pereboom D, Vargas M (2007). Revisionism. Four views on free will.

[CR55] Wade N (1978). Guillemin and schally: A race spurred by rivalry. Science.

[CR56] Wade N (1978). Guillemin and schally: the three-lap race to stockholm. Science.

[CR57] Wainwright M (1987). The history of the therapeutic use of crude penicillin. Medical History.

[CR58] Wallace JR (1994). Responsibility and the moral sentiments.

[CR59] Watson G, Watson G (2004). Two faces of responsibility. Agency and answerability.

[CR60] Zimmerman MJ (2002). Taking luck seriously. Journal of Philosophy.

[CR61] Zuckerman H (1970). Stratification in American science. Sociological Inquiry.

[CR62] Zuckerman H (1996). Scientific elites. Nobel laureates in the United States.

